# Combination Therapy of Ledipasvir and Itraconazole in the Treatment of COVID-19 Patients Coinfected with Black Fungus: An *In Silico* Statement

**DOI:** 10.1155/2022/5904261

**Published:** 2022-04-19

**Authors:** Supriyo Saha, Gyu Seong Yeom, Satish Balasaheb Nimse, Dilipkumar Pal

**Affiliations:** ^1^School of Pharmaceutical Sciences & Technology, Sardar Bhagwan Singh University, Dehradun, 248161 Uttarakhand, India; ^2^Institute of Applied Chemistry and Department of Chemistry, Hallym University, Chuncheon 24252, Republic of Korea; ^3^Department of Pharmaceutical Sciences, Guru Ghasidas Vishwavidyalaya (A Central University), C.G, 495009, Bilaspur, India

## Abstract

The manuscript mainly aimed at providing clues on improving the innate immunity of coronavirus patients and safeguarding them from both new mutant strains and black fungus infections. Coronavirus is readily mutating from one variant to another. Among the several variants, we selected SARS-CoV-2 B.1.1.7 in this study. Upon infection of any virus, ideally, the phagocytic cells of the host engulf and destroy the virus by a mechanism called phagocytosis. However, compromised immunity impairs phagocytosis, and thus, restoring the immune system is crucial for a speedy recovery of infected patients. The autophagy and activation of Toll-like receptor-4 are the only ways to restore innate immunity. Recently, immunocompromised COVID-19 patients have been suffering from the coinfection of black fungus. Rhizomucor, a black fungus species, causes more than 75% of cases of mucormycosis. Here, we present the results of molecular docking studies of sixty approved antiviral drugs targeting receptors associated with the SARS-CoV-2 B 1.1.7 variant (PDB id: 7NEH), activating the innate immune system (PDB id: 5YEC and 5IJC). We also studied the twenty approved antifungal drugs with *Rhizomucor miehei* lipase propeptide (PDB id: 6QPR) to identify the possible combination therapy for patients coinfected with coronavirus and black fungus. The ledipasvir showed excellent docking interactions with the 7NEH, 5YEC, and 5IJC, indicating that it is a perfect candidate for the treatment of COVID-19 patients. Itraconazole showed significant interaction with 6QPR of *Rhizomucor miehei*, suggesting that itraconazole can treat black fungus infections. In conclusion, the combination therapy of ledipasvir and itraconazole can be a better alternative for treating COVID-19 patients coinfected with black fungus.

## 1. Introduction

Severe acute respiratory syndrome coronavirus 2 (SARS-CoV-2) infection that causes coronavirus disease 2019 (COVID-19) has threatened public health worldwide [1, 2]. It is almost a year and a half after the COVID-19 pandemic outbreak, and we are still struggling to find optimum drug therapy [3]. The emergence of the SARS-CoV-2 variants has made the situation hard to control [4, 5]. Even though the current vaccines against SARS-CoV-2 prevent severe complications and deaths effectively, the treatment options are still under validation [6, 7]. Recently, immunocompromised COVID-19 patients have been suffering from the coinfection of black fungus (mucormycosis) [8, 9]. COVID-19 patients with a compromised immune system or having diabetic ketoacidosis are highly prone to mucormycosis [10–13]. Rhizomucor, a black fungus species, causes more than 75% of cases of mucormycosis [14, 15]. Mucormycosis infection is directly linked with *Rhizopus* and *Aspergillus* species (*Rhizopus oryzae* and *Aspergillus oryzae*) [16–20]. The cases of COVID-19-associated mucormycosis have been increasing worldwide since early 2021 [21], and several of such pateints have died in India [22, 23]. Therefore, drug treatment options for the combination therapy of COVID-19 patients coinfected with black fungus are urgently needed to mitigate the effects on public health and the economy. In this scenario, drug repurposing is the quickest method for identifying possible drug candidates and their implementation in the treatment [24–27].

One of the applications of drug repurposing is to identify existing drugs that can treat other diseases [28–30]. The advantages of drug repurposing over conventional drug discovery approaches include low development costs, shorter drug development timelines, no phase 1 clinical trial requirement, and potential for reuse [31, 32]. According to a recent report, each drug can interact with three targets, and each target has about 4.7 drugs, indicating that polypharmacology is a common phenomenon and basis for repurposing drugs [33]. Thus, computer-aided drug design (CADD) and polypharmacology allow rational identification of an FDA-approved drug for pursuing targets other than previously established ones. Therefore, we proposed the CADD-based identification of FDA-approved drugs for the combination therapy of COVID-19 patients coinfected with the black fungus.

SARS-CoV-2 is readily mutating from one variant to another. Among the several variants, we selected SARS-CoV-2 B.1.1.7 in this study and targeted the receptor-binding domain of SARS-CoV-2 spike glycoprotein (PDB 7NEH [34, 35]. Upon infection, ideally, the phagocytic cells of the host engulf and destroy the virus by a mechanism called phagocytosis [36–38]. However, in compromised immunity, autophagy and activation of Toll-like receptor-4 (TLR4) are the only ways to restore innate immunity [39, 40]. The agonistic activity of TLR4 subsequently activates the NF-K*β* (nuclear factor kappa-light-chain-enhancer of activated B cells) linked inflammatory pathways along with increased cytokine production and restores the innate immunity system [41, 42]. Therefore, we selected two other target proteins related to autophagy (PDB 5YEC) [43] and TLR4 (PDB 5IJC) [42] for this study. Finally, we chose the target receptor protein (PDB 6QPR) [44] associated with *Rhizomucor miehei* to tackle the black fungus coinfections in COVID-19 patients.

Herein, we present the results of molecular docking studies performed using sixty approved antiviral drugs targeting glycoprotein (PDB 7NEH) associated with SARS-CoV-2, receptors related to autophagy (PDB 5YEC), and TLR4 (PDB 5IJC). We also studied the reaction of twenty approved antifungal drugs with the target protein (PDB 6QPR) associated with *Rhizomucor miehei*. The ligand−target interaction profile and binding free energy allowed us to identify ledipasvir and itraconazole as possible candidates for the mono or combination therapy in COVID-19 patients coinfected with black fungus.

## 2. Methods

### 2.1. FDA-Approved Antiviral and Antifungal Drugs

The FDA-approved drugs are known for their function and toxic effects on humans. Hence, these drugs are generally used in drug-repurposing studies to identify a promising candidate for a new target. In this study, our first target was to identify an FDA-approved antiviral drug that can bind to the SARS-CoV-2 spike glycoprotein, proteins related to autophagy, and TLR4. Therefore, we selected about sixty antiviral drugs (see the supporting information Table S1). These drugs are approved to treat several viral infections, including the diseases caused by human immunodeficiency virus, hepatitis C virus, human influenza virus, respiratory syncytial virus, herpes simplex virus, hepatitis B virus, and human smallpox. The selected antiviral drugs target various enzymes and proteins, including protease, reverse transcriptase, integrase, envelope glycoprotein GP120, NS5B polymerase, RNA polymerase, and DNA polymerase VP37 envelope wrapping protein. The second target of this study was to identify an antifungal drug that can be used to treat black fungus infections. Therefore, we selected easily available twenty antifungal FDA-approved medicines (see the supporting information Table S2).

### 2.2. Computational Procedures

#### 2.2.1. Ligand Preparation

The antiviral drugs studied here include amprenavir (AMP), atazanavir (ATA), darunavir (DAR), fosamprenavir (FOS), indianvir (IND), lopinavir (LOP), nelfinavir (NEL), ritonavir (RIT), saquinavir (SAQ), tipranavir (TIP), delaviridine (DEL), doravirine (DOR), efavirenz (EFA), etravirine (ETA), nevirapine (NEV), rilpivirine (RIL), abacavir (ABA), didanosine (DID), emtricitabine (EMT), lamivudine (LAM), stavudine (STA), tenofovir (TEN), zidovudine (ZID), bictegravir (BIC), dolutegravir (DOL), elvitegravir (ELV), raltegravir (RAL), fostemsavir (FOS), danoprevir (DAN), grazoprevir (GRA), paritaprevir (PAR), simeprevir (SIM), daclatasvir (DAC), ledipasvir (LED), ombitasvir (OMB), elbasvir (ELB), velpatasvir (VEL), pibrentasvir (PIB), sofosbuvir (SOF), dasabuvir (DIS), Baloxavir marboxil (BAL), favipiravir (FAV), laninamivir (LAN), oseltamivir (OSE), peramivir (PER), zanamivir (ZAN), ribavirin (RIB), aciclovir (ACI), brivudine (BRI), famciclovir (FAM), idoxuridine (IDO), penciclovir (PEN), valaciclovir (VAL), cidofovir (CID), foscarnet (FOS), ganciclovir (GAN), adefovir (ADE), besifovir (BES), clevudine (CLE), and tecovirimat (TEC). The antifungal drugs included amphotericin (AMP), Anidulagin (ANI), caspofungin (CAS), econazole (ECO), fluconazole (FLU), isavuconazole (ISA), itraconazole (ITR), luliconazole (LUL), micafungin (MIC), miconazole (MIZ), nystatin (NYS), posaconazole (PSA), voriconazole (VOR), ipconazole (IPC), isoconazole (ISO), sulconazole (SUL), terconazole (TER), tioconazole (TIO), clotrimazole (CLO), and terbinafine (TER). We used Avogadro 1.2 software [45] to draw the molecular structures of ligands, including 60 antiviral drugs and 20 antifungal drugs, using their smile notifications obtained from Pubchem. Then, these structures were optimized using Gaussian 09 at the DFT/B3LYP/6-31G(d) level and used in the docking experiments.

#### 2.2.2. Protein Preparation

We conducted the docking studies using the reported crystal structure of SARS-CoV-2 spike glycoprotein (PDB 7NEH, resolution 1.77 Å), protein related to autophagy (PDB 5YEC, resolution 2.15 Å), TLR4 (PDB 5IJC, resolution 2.57 Å), and a fungal protein (PDB 6QPR, resolution 1.45 Å). We obtained the crystal structures of studied proteins from the Protein Data Bank (http://www.rcsb.org/pdb/home/home.do). Structures of all proteins were prepared in AutoDockTools by eliminating the water molecules, assigning ADT atom types, adding polar hydrogen atoms, and adding Kollman charges [46].

#### 2.2.3. Molecular Docking and Molecular Dynamic Simulation

We performed the blind docking procedures according to the previous reports [47–49]. Here, we used AutoDock 4.2.6 software to determine the binding abilities of antiviral drugs to proteins 7NEH, 5YEC, and 5IJC prepared as mentioned above. Similarly, we docked the antifungal drugs with 6QPR. The Lamarckian genetic algorithm (LGA) with local search was applied to seek the best binding site of ligands in respective proteins. Protein-ligand docking default parameters with the grid box dimensions are listed below. About 25 conformations were set as output for the ligand-protein docking experiments. The conformations with the lowest binding energy in each case were selected. Finally, the docking results were visualized and analyzed using BIOVIA Discovery Studio Visualizer 4.5. [50]. The protein-ligand docking default parameters and grid box grid measurement values for 7NEH (center_x = 1.903, center_y = 72.062, center_z = 16.274. size_x = 24, size_y = 24, size_z =24, exhaustiveness = 8. ASN 343 was considered as flexible residue), 5YEC (center_x = 10.544, center_y = −31.442, center_z = −18.62. size_x = 24, size_y = 24, size_z = 24, exhaustiveness = 8. ASP 355 was considered as flexible residue), 5IJC (center_x = 30.421, center_y = −13.194, center_z = 28.525. size_x = 24, size_y = 24, size_z = 24, exhaustiveness = 8. SER 413 was considered as flexible residue), and 6QPR (center_x = 16.325, center_y = 63.778, center_z = −8.601. size_x = 24, size_y = 24, size_z =24, exhaustiveness = 8. ASP 72 was considered as flexible residue) were used in the docking experiments. The molecular dynamic simulation of the protein-ligand complexes was performed using VMD-NAMD software with CHARMM forcefield and the 10 Å water layer parameters in all directions [51]. A total of 100,000 cycles of time steps were performed.

## 3. Results and Discussions

Verifying the protein structure is vital during an effective structure-based drug design. One of the protein structure verification methods is to analyze Ramachandran's plot. [52] Therefore, we checked the quality of all four crystal structures of proteins (PDB 7NEH, 5YEC, 5IJC, and 6QPR) used in this study with the PROCHECK server that generates Ramachandran's plot [53]. Ramachandran's plots (see the supporting information Figure S1-S4) were analyzed. Ramachandran's plot for SARS-CoV-2 spike glycoprotein (PDB 7NEH) demonstrated that amino acid residues are categorically distributed in the favorable (89.6%), additional allowed region (9.9%), generously allowed (0.4%), and disallowed regions (0.2%) (Figure S1). Similarly, Ramachandran's plot of protein related to the autophagy (PDB 5YEC) demonstrated that amino acid residues are distributed in the favorable (92.1%), additional allowed region (7.7%), and disallowed regions (0.1%) (Figure S2). Ramachandran's plot of TLR4 (PDB 5IJC) revealed that about a total of 99.9% residues fall in favorable and additional allowed regions. Whereas, only few residues lie in generously allowed (0.2%) and disallowed regions (0.3%) (Figure S3). The fungal protein (PDB 6QPR) amino acid residue distribution in the favorable region is 90.8%, additional allowed region is 8.5%, and disallowed regions is 0.7% (Figure S3). It is important to note that none of the residue for this protein was found in the disallowed region. These results explicitly back the quality and reliability of the studied protein structures. Hence, these structures were used for further study.

**Figure 1:**
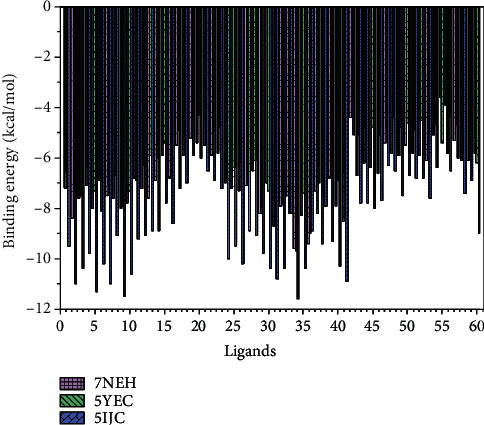
Binding interactions of sixty antiviral compounds with the SARS-CoV-2 spike glycoprotein (PDB 7NEH), protein related to the autophagy (PDB 5YEC), and TLR4 (PDB 5IJC). 1, AMP; 2, ATA; 3, DAR; 4, FOS; 5, IND; 6, LOP; 7, NEL; 8, RIT; 9, SAQ; 10, TIP; 11, DEL; 12, DOR; 13, EFA; 14, ETA; 15, NEV; 16, RIL; 17, ABA; 18, DID; 19, EMT; 20, LAM; 21, STA; 22, TEN; 23, ZID; 24, BIC; 25, DOL; 26, ELV; 27, RAL; 28, FOS; 29, DAN; 30, GRA; 31, PAR; 32, SIM; 33, DAC; 34, LED; 35, OMB; 36, ELB; 37, VEL; 38, PIB; 39, SOF; 40, DAS; 41, BAL; 42, FAV; 43, LAN; 44, OSE; 45, PER; 46, ZAN; 47, RIB; 48, ACI; 49, BRI; 50, FAM; 51, IDO; 52, PEN; 53, VAL; 54, CID; 55, FOS; 56, GAN; 57, ADE; 58, BES; 59, CLE; 60, TEC.

The molecular docking results of the studied sixty antiviral compounds with the SARS-CoV-2 spike glycoprotein (PDB 7NEH), a protein related to autophagy (PDB 5YEC), and TLR4 (PDB 5IJC) are presented in Figure 1 (see the supporting information Table S3-S5).

As shown in Figure 1 and Table S3, the ledipasvir (−9.6 Kcal/mol), elbasvir (−9.4 Kcal/mol), and pibrentasvir (−9.4 Kcal/mol) exhibited higher docking interaction energies with SARS-CoV-2 spike glycoprotein (PDB 7NEH). Among other antiviral drugs, paritaprevir (−8.7 Kcal/mol), baloxavir marboxil (−8.5 Kcal/mol), danoprevir (−8.2 Kcal/mol), saquinavir (−8.0 Kcal/mol), and indinavir (−8.0 Kcal/mol) also showed good docking energies. The molecular docking interaction outcomes between protein related to autophagy (PDB 5YEC), and sixty antiviral drugs showed that ledipasvir (−9.7 Kcal/mol) and elbasvir (−9.0 Kcal/mol) have strong docking interaction energies (Figure 1 and Table S4). The paritaprevir (−8.7 Kcal/mol), velpatasvir (−8.2 Kcal/mol), daclatasvir (−8.2 Kcal/mol), and lopinavir (−8.1 Kcal/mol) were moderately docked with this receptor as indicated by low docking energies.

As shown in Figure 1 and Table S5, the docking experiments of sixty antiviral drugs with the TLR4 (PDB 5IJC) showed that ledipasvir (−11.6 Kcal/mol) and saquinavir (−11.5 Kcal/mol) could bind this protein with the strong interactions indicated by high binding energies. Further, the other antiviral drugs such as indinavir (−11.3 Kcal/mol), atazanavir (−11.0 Kcal/mol), nelfinavir (−11.0 Kcal/mol), paritaprevir (−10.8 Kcal/mol), tipranavir (−10.6 Kcal/mol), grazoprevir (−10.4 Kcal/mol), simeprevir (−10.4 Kcal/mol), ombitasvir (−10.2 Kcal/mol), lopinavir (−10.2 Kcal/mol), and elvitegravir (−10.2 Kcal/mol) showed moderate docking energies.

**Figure 2:**
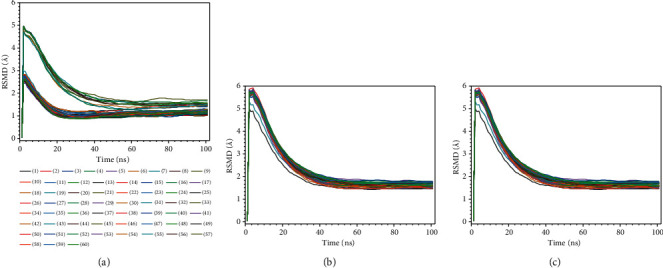
Plots of root-mean-square deviations of each protein-ligand complexes of sixty antiviral compounds with the (a) SARS-CoV-2 spike glycoprotein (PDB 7NEH), (b) protein related to the autophagy (PDB 5YEC), and (c) TLR4 (PDB 5IJC). 1, AMP; 2, ATA; 3, DAR; 4, FOS; 5, IND; 6, LOP; 7, NEL; 8, RIT; 9, SAQ; 10, TIP; 11, DEL; 12, DOR; 13, EFA; 14, ETA; 15, NEV; 16, RIL; 17, ABA; 18, DID; 19, EMT; 20, LAM; 21, STA; 22, TEN; 23, ZID; 24, BIC; 25, DOL; 26, ELV; 27, RAL; 28, FOS; 29, DAN; 30, GRA; 31, PAR; 32, SIM; 33, DAC; 34, LED; 35, OMB; 36, ELB; 37, VEL; 38, PIB; 39, SOF; 40, DAS; 41, BAL; 42, FAV; 43, LAN; 44, OSE; 45, PER; 46, ZAN; 47, RIB; 48, ACI; 49, BRI; 50, FAM; 51, IDO; 52, PEN; 53, VAL; 54, CID; 55, FOS; 56, GAN; 57, ADE; 58, BES; 59, CLE; 60, TEC.

We studied the molecular dynamic stability of each protein-ligand complex of sixty antiviral compounds with the SARS-CoV-2 spike glycoprotein (PDB 7NEH), a protein related to autophagy (PDB 5YEC), and TLR4 (PDB 5IJC). The results of molecular dynamic simulation in terms of root-mean-square deviation (RMSD) fluctuations and simulation time are presented in Figure 2. The least-squares fitted RMSDs of the studied antiviral agents are in the range of 1.09−2.20 Å for binding interaction with the SARS-CoV-2 spike glycoprotein (PDB 7NEH). The RMSDs for binding interaction of the studied antiviral agents with the protein related to autophagy (PDB 5YEC) were found to be in the range of 1.56−2.46 Å. At the same time, these values were in the range of 1.46−1.77 Å for the binding interactions of studied ligands with the TLR4 (PDB 5IJC). These results indicate that the molecular dynamic trajectories are overall stable for all the studied protein-ligand complexes.

Among the sixty antiviral compounds, the top three drugs that showed strong binding interactions with SARS-CoV-2 spike glycoprotein (PDB 7NEH) were ledipasvir, elbasvir, and pibrentasvir (Table S3). The top three drugs that showed higher binding interactions with protein related to autophagy (PDB 5YEC) were ledipasvir, elbasvir, and paritaprevir (Table S4). In contrast, the top three ligands that showed high binding energy with TLR4 (PDB 5IJC) were ledipasvir, saquinavir, and indinavir (Table S5).

**Figure 3:**
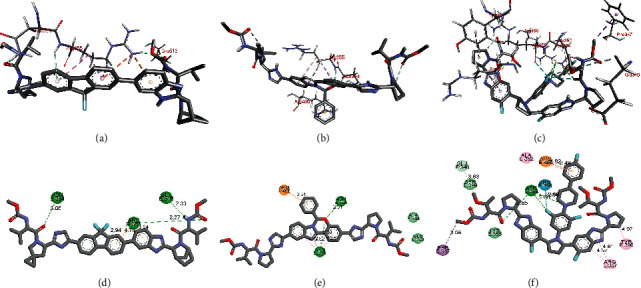
Binding interactions of ledipasvir (a) and (b), elbasvir (c) and (d), and pibrentasvir (e) and (f) with SARS-CoV-2 spike glycoprotein (PDB 7NEH).

As shown in Figures 3(a) and 3(b), ledipasvir demonstrates the hydrogen bonding interactions with ASN 354, GLU 516, and ARG 355 in the cavity of SARS-CoV-2 spike glycoprotein (PDB 7NEH). The *π* − *π* stacking interaction with ARG 355 was also observed. Elbasvir demonstrated two hydrogen binding interactions, each with ASN 354 and ARG 355 (Figures 3(c) and 3(d)). Further, the *π* − *π* stacking interactions with ARG 466 and ARG 355 were also observed in a docked structure of elbasvir SARS-CoV-2 spike glycoprotein (PDB 7NEH). As shown in Figures 3(e) and 3(f), the docked structure of pibrentasvir in the binding site of SARS-CoV-2 spike glycoprotein showed two hydrogen-bonding interactions (with ASN 354 and ARG 355) and two *π* − *π* stacking interactions (with ARG 466 and PHE 347).

**Figure 4:**
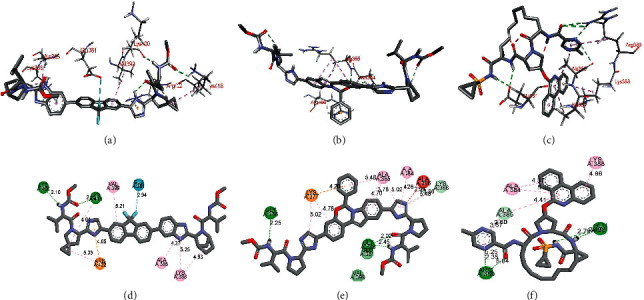
Binding interactions of ledipasvir (a) and (b), elbasvir (c) and (d), and paritaprevir (e) and (f) with protein related to autophagy (PDB 5YEC).

The molecular docking study indicated that the drugs ledipasvir, elbasvir, and paritaprevir show superior binding affinity with protein related to autophagy (PDB 5YEC) (Table S4). Therefore, the top binding poses of these drugs in the binding site of protein related to autophagy (PDB 5YEC) are presented in Figure 4. As depicted in Figures 4(a) and 4(b), ledipasvir interacts with LYS 400 and LYS 418 by hydrogen bonding as hydrogen bond donor and acceptor. The amino acids ALA 385, LYS 388, and VAL 399 were found to show a *π*-alkyl interaction with the ledipasvir. It is important to note that ARG 422 showed a cation-*π* interaction with the imidazole ring of ledipasvir. No doubt, the compound effect of these interactions resulted in the topmost binding energy of −9.7 (RMSD = 2.213 Å) for ledipasvir upon docking with PDB 5YEC.

On the other hand, as shown in Figures 4(c) and 4(d), elbasvir also demonstrated at least three hydrogen binding interactions with the amino acids GLY 376, GLU 381, and GLY 398. A cation-*π* interaction with LYS 377 and *π*-alkyl interactions with ALA 385 and ALA 384 were also observed. However, elbasvir's binding energy of −9.0 (RMSD = 2.231 Å) was less than for ledipasvir (Table S4). The unfavorable interaction between the ALA 396 and one of the imidazole ring of elbasvir might be the reason behind the decreased binding efficiency of elbasvir compared to ledipasvir. As shown in Figures 4(e) and 4(f), paritaprevir was found to show hydrogen bonding interactions with GLU 381 and ARG 389. Besides these interactions, the amino acids ALA 384 and LYS 388 were found to establish a *π*-alkyl interaction with the paritaprevir. As a result, paritaprevir demonstrated binding energy of −8.7 (RMSD = 1.558 Å) upon docking with PDB 5YEC. These results indicate the top-ranked antiviral drug that has a higher potential to bind with PDB 5YEC is ledipasvir.

The molecular docking studies to identify the top-ranked ligand for TLR4 (PDB 5IJC) revealed that ledipasvir, saquinavir, and indinavir were the top three drugs with superior binding affinity among studied antiviral agents (Table S5).

**Figure 5:**
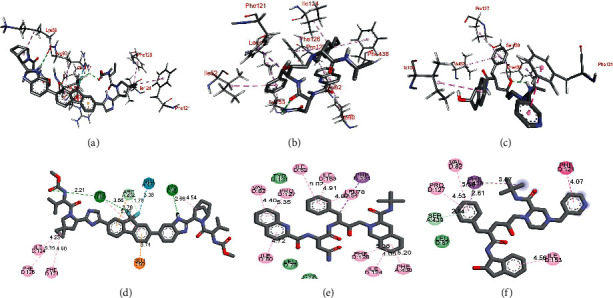
Binding interactions of ledipasvir (a) and (b), saquinavir (c) and (d), and indinavir (e) and (f) with TLR4 (PDB 5IJC).

As depicted in Figures 5(a) and 5(b), ledipasvir interacts with ARG 90 and LYS 89 using hydrogen bonding and with the amino acids PHE 121, ILE 124, and PHE 126 by Van der Walls interaction in the binding pocket of TLR4 (PDB 5IJC). The GLU 92 demonstrates a cation-*π* interaction with the fluorine nucleus of ledipasvir. The calculated binding energy for the interaction of ledipasvir with TLR4 was found to be −11.6 (RMSD = 1.584 Å). Similarly, as shown in Figures 5(c) and 5(d), the molecular docking studies for binding of saquinavir with TLR4 (PDB 5IJC) revealed the sole Van der Walls interactions with amino acids ILE 52, VAL 82, ILE 124, PHE 126, PRO 127, ILE 153, and PHE 438 leading to the binding energy of −11.5 (RMSD = 1.592 Å). Finally, the interaction of indinavir in the binding pocket of TLR4 (PDB 5IJC) was mostly Van der Waals type with the amino acids VAL 82, PHE 121, PRO 127, ILE 153, PHE 438, and SER 439, as depicted in Figures 5(e) and 5(f). The binding energy for the indinavir TLR4 (PDB 5IJC) complex was found to be −11.3 (RMSD = 1.621 Å).

The molecular docking studies of sixty antiviral agents revealed that the ledipasvir demonstrates higher binding interactions with all three proteins 7NEH (−9.6 Kcal/mol), 5YEC (−9.7 Kcal/mol), and 5IJC (−11.6 Kcal/mol). Therefore, this study reveals that ledipasvir can be used to treat COVID-19. A further clinical study using ledipasvir is inevitable for its clinical application to treat patients infected with SARS-CoV-2.

**Figure 6:**
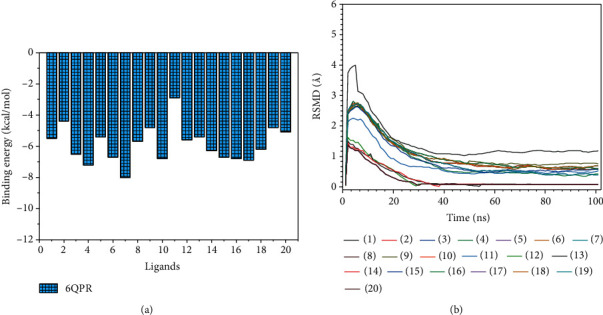
(a) Binding interactions and (b) plots of root-mean-square deviations of protein-ligand complexes of twenty antifungal compounds with fungal protein (PDB 6QPR). 1, AMP; 2, ANI; 3, CAS; 4, ECO; 5, FLU; 6, ISA; 7, ITR; 8, LUL; 9, MIC; 10, MIZ; 11, NYS; 12, PSA; 13, VOR; 14, IPC; 15, ISO; 16, SUL; 17, TER; 18, TIO; 19, CLO; 20, TER.

We also evaluated the respective binding energies of about 20 antifungal agents against the fungal protein (PDB 6QPR) to identify the suitable drug that can be used in combination with ledipasvir for the treating COVID-19 patients coinfected with black fungus (Table S6). As shown in Figure 6(a), we found that the itraconazole (– 8.0 Kcal/mol (RMSD =1.923 Å)) and econazole (– 7.2 Kcal/mol (RMSD =2.102 Å)) demonstrated higher binding energies compared to other antifungal agents. The results of molecular dynamic simulation in terms of RMSD fluctuations and simulation time are presented in Figure 6(b). The RMSDs of the studied antifungal agents for binding interaction with fungal protein (PDB 6QPR) were 0.042–3.74 Å. These results indicate that the molecular dynamic trajectories are overall stable for all the studied protein-ligand complexes.

**Figure 7:**
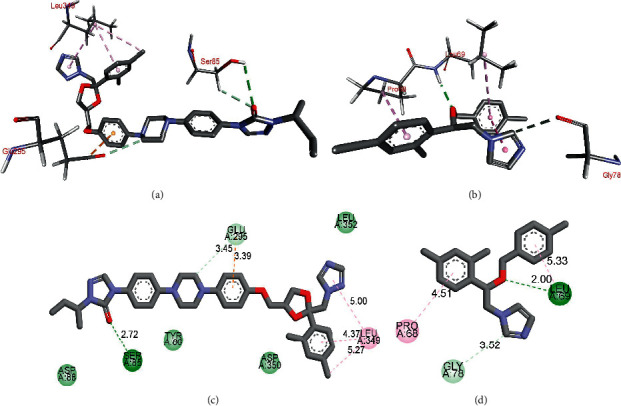
Binding interactions of itraconazole (a) and (b) and econazole (c) and (d) with fungal protein (PDB 6QPR).

As shown in Figures 7(a) and 7(b), itraconazole interacts with the binding site of fungal protein (PDB 6QPR) using hydrogen binding with SER 85. Further, the cation-*π* interaction with GLU 295 and *π*-alkyl interactions with LEU 349 were also observed. Whereas econazole mainly showed hydrogen binding with LEU 69 and *π*-alkyl interaction with PRO 68, as shown in Figures 7(c) and 7(d). It is evident from these results that itraconazole has a higher chance to target the fungal protein (PDB 6QPR).

The outbreaks of highly infectious diseases, such as COVID-19, warrant immediate identification of an optimum drug therapy due to the unavailability of the known medicine. The drug repurposing based on the computational methods and computer-aided drug discovery platforms offer higher chances for a drug's success than blindly treating the diseases such as COVID-19 to decrease the chances of mutated versions further. Computational methods such as molecular docking studies offer possible treatment options within a short period.

We have screened about sixty antiviral agents and twenty antifungal agents in the present work, searching for possible treatment options for the patients coinfected with SARS-CoV-2 and black fungus. The study revealed that antihepatitis-C drugs such as ledipasvir, elbasvir, and pibrentasvir can effectively bind with a new variant of coronavirus SARS-CoV-2 B 1.1.7 and SARS-CoV-2 protein (PDB 7NEH). The present study results are in concordance with the previous report. [54, 55] Previously, it was reported that sofosbuvir showed effective binding interaction with the protease (PDB 6 LU7) of the initial coronavirus strain. However, in the current study, using the variant protein, we found that the sofosbuvir showed lower binding energy of –6.8 (RMSD = 1.220 Å) than the ledipasvir.

Further, the antihepatitis-C drugs ledipasvir and elbasvir also showed higher binding energies for the interaction with the autophagy-related receptor (PDB 5YEC). Hence, we believe that treatment with ledipasvir in COVID-19 can activate our innate immunity. The TLR4 receptor (PDB 5IJC) studied here can bind to neoseptin-3, which is an agonist of TLR4 and myeloid differentiation factor [56]. It is reported that most antihepatitis-C and anti-HIV drugs showed similar activity as neoseptin-3 [57, 58]. Therefore, we believe that the ledipasvir can effectively activate TLR4.

Though mucormycosis is an infrequent infection, it is widely spread in COVID-19 patients. The overuse of immunosuppressive agents caused the sudden outbreak of black fungus infection. Amphotericin B is highly accepted for the treatment of black fungus. However, a reported study showed that amphotericin B has a higher risk for renal failure. Therefore, an effective drug therapy against mucormycosis that can be used with the COVID-19 drug is the need of the hour [59, 60]. Here, our initial results on the molecular docking of twenty different antifungal agents with the fungal protein (PDB 6QPR) originated from the *Rhizomucor miehei* revealed that the itraconazole is an excellent candidate. Hence, according to a computational study presented here, ledipasvir and itraconazole can be effective treatment options for COVID-19 patients coinfected with black fungus.

## 4. Conclusion

In this study, we took advantage of the reported crystal structure of a new variant of SARS-CoV-2 spike glycoprotein (PDB 7NEH) and the crystal structures of protein related to autophagy (PDB 5YEC), TLR4 (PDB 5IJC), and a fungal protein (PDB 6QPR). We used these crustal structures to conduct multiscale drug repurposing screenings to identify the effective treatment options for COVID-19 patients coinfected with black fungus. The present study suggests that the drug repurposing screening based on computational methods is very efficient, and it can help identify the potential drug candidates in a short time. The results presented in this study indicate that the ledipasvir can inhibit the SARS-CoV-2 spike glycoprotein and interact with the protein related to autophagy (PDB 5YEC) and TLR4 (PDB 5IJC). Hence, ledipasvir can exhibit multifaceted synergistic drug action to treat COVID-19. Identifying itraconazole for the treatment of black fungus allows us to presume that it can be used in combination with ledipasvir to treat COVD-19 patients coinfected with black fungus.

## Data Availability

All data used to support the findings of this study are included within the main text and in the Supporting Information file.

## Supplementary Materials

Supplementary Materials Table S1: list of the antiviral drugs (*n* = 60) used in this study and their original targets. Table S2: list of the antifungal drugs (*n* = 20) used in this study. Figure S1: Ramachandran plot for SARS-CoV-2 spike glycoprotein (PDB 7NEH, resolution 1.77 Å). Figure S2: Ramachandran plot for proteins related to the autophagy (PDB 5YEC, resolution 2.15 Å). Figure S3: Ramachandran plot for TLR4 (PDB 5IJC, resolution 2.57 Å). Figure S4: Ramachandran plot for fungal protein (PDB 6QPR, resolution 1.45 Å). Table S3: molecular docking binding energies of antiviral drugs with SARS-CoV-2 spike glycoprotein (PDB 7NEH). Table S4: molecular docking binding energies of antiviral drugs with autophagy receptor (PDB 5YEC). Table S5: molecular docking binding energies of antiviral drugs with TLR4 (PDB 5IJC). Table S6: molecular docking binding energies of antifungal drugs with target protein of *Rhizomucor miehei* (PDB 6QPR).
