# Supplementary material for: Combination Therapy of Ledipasvir and Itraconazole in the Treatment of COVID-19 Patients Coinfected with Black Fungus: An In Silico Statement
Source: Biomed Res Int. 2022 Apr 19;2022:5904261. doi: 10.1155/2022/5904261 (PMC9020143; doi:10.1155/2022/5904261)
Supplement: Supplementary Materials — Table S1: list of the antiviral drugs (n = 60) used in this study and their original targets. Table S2: list of the antifungal drugs (n = 20) used in this study. Figure S1: Ramachandran plot for SARS-CoV-2 spike glycoprotein (PDB 7NEH, resolution 1.77 Å). Figure S2: Ramachandran plot for proteins related to the autophagy (PDB 5YEC, resolution 2.15 Å). Figure S3: Ramachandran plot for TLR4 (PDB 5IJC, resolution 2.57 Å). Figure S4: Ramachandran plot for fungal protein (PDB 6QPR, resolution 1.45 Å). Table S3: molecular docking binding energies of antiviral drugs with SARS-CoV-2 spike glycoprotein (PDB 7NEH). Table S4: molecular docking binding energies of antiviral drugs with autophagy receptor (PDB 5YEC). Table S5: molecular docking binding energies of antiviral drugs with TLR4 (PDB 5IJC). Table S6: molecular docking binding energies of antifungal drugs with target protein of Rhizomucor miehei (PDB 6QPR). [file 5904261.f1.docx]

**Combination therapy of ledipasvir and itraconazole in the treatment of COVID-19 patients co-infected with black fungus: An *In-silico* Statement**

**Supriyo Saha^2,4^, Yeom Gyu Seong^1,4^, Satish Balasaheb Nimse^1*^, Dilipkumar Pal^3*^**

^1^Institute of Applied Chemistry and Department of Chemistry, Hallym University, Chuncheon 200702, Republic of Korea.

^2^School of Pharmaceutical Sciences & Technology, Sardar Bhagwan Singh University, Dehradun-248161, Uttarakhand, India.

^3^Department of Pharmaceutical Sciences, Guru Ghasidas Vishwavidyalaya (A Central University), Bilaspur, C.G., 495 009, India.

^4^These authors contributed equally. Hence both should be considered as first authors

**Supporting information**

**Table S1.** List of the antiviral drugs (n = 60) used in this study and their original targets

| **Sr. No.** | **Antiviral drugs** | **Target enzymes** |
| --- | --- | --- |
| 1. | Amprenavir | Protease Human Immunodeficiency Virus |
| 2. | Atazanavir |  |
| 3. | Darunavir |  |
| 4. | Fosemprenavir |  |
| 5. | Indianvir |  |
| 6. | Lopinavir |  |
| 7. | Nelfinavir |  |
| 8. | Ritonavir |  |
| 9. | Saquinavir |  |
| 10. | Tipranavir |  |
| 11. | Delaviridine | Reverse transcriptase Human Immunodeficiency Virus |
| 12. | Doravirine |  |
| 13. | Efavirenz |  |
| 14. | Etavirine |  |
| 15. | Nevirapine |  |
| 16. | Rilpivirine |  |
| 17. | Abacavir |  |
| 18. | Didanosine |  |
| 19. | Emtricitabine |  |
| 20. | Lamivudine |  |
| 21. | Stavudine |  |
| 22. | Tenofovir |  |
| 23. | Zidovudine |  |
| 24. | Bictegravir | Integrase Human Immunodeficiency Virus |
| 25. | Dolutegravir |  |
| 26. | Elvitegravir |  |
| 27. | Raltegravir |  |
| 28. | Fostemsavir | Envelope glycoprotein gp120 Human Immunodeficiency Virus |
| 29. | Danoprevir | NS5B polymerase Hepatitis C Virus |
| 30. | Grazoprevir |  |
| 31. | Paritaprevir |  |
| 32. | Simeprevir |  |
| 33. | Daclatasvir |  |
| 34. | Ledipasvir |  |
| 35. | Ombitasvir |  |
| 36. | Elbasvir |  |
| 37. | Velpatasvir |  |
| 38. | Pibrentasvir |  |
| 39. | Sofosbuvir |  |
| 40. | Dasabuvir |  |
| 41. | Baloxavir marboxil | RNA polymerase Human Influenza Virus |
| 42. | Favipiravir |  |
| 43. | Laninamivir | Neuraminidase Human Influenza Virus |
| 44. | Oseltamivir |  |
| 45. | Peramivir |  |
| 46. | Zanamivir |  |
| 47. | Ribavirin | RNA polymerase Respiratory syncytial virus |
| 48. | Aciclovir | DNA polymerase Herpes simplex virus |
| 49. | Brivudine |  |
| 50. | Famciclovir |  |
| 51. | Idoxuridine |  |
| 52. | Penciclovir |  |
| 53. | Valaciclovir |  |
| 54. | Cidofovir |  |
| 55. | Foscarnet |  |
| 56. | Ganciclovir |  |
| 57. | Adefovir | DNA polymerase Hepatitis B virus |
| 58. | Besifovir |  |
| 59. | Clevudine |  |
| 60. | Tecovirimat | VP37 envelope wrapping protein Human smallpox |

**Table S2.** List of the antifungal drugs (n = 20) used in this study

| **Sr. No.** | **Antifungal drugs** |
| --- | --- |
| 1. | Amphotericin |
| 2. | Anidulagin |
| 3. | Caspofungin |
| 4. | Econazole |
| 5. | Fluconazole |
| 6. | Isavuconazole |
| 7. | Itraconazole |
| 8. | Luliconazole |
| 9. | Micafungin |
| 10. | Miconazole |
| 11. | Nystatin |
| 12. | Posaconazole |
| 13. | Voriconazole |
| 14. | Ipconazole |
| 15. | Isoconazole |
| 16. | Sulconazole |
| 17. | Terconazole |
| 18. | Tioconazole |
| 19. | Clotrimazole |
| 20. | Terbinafine |


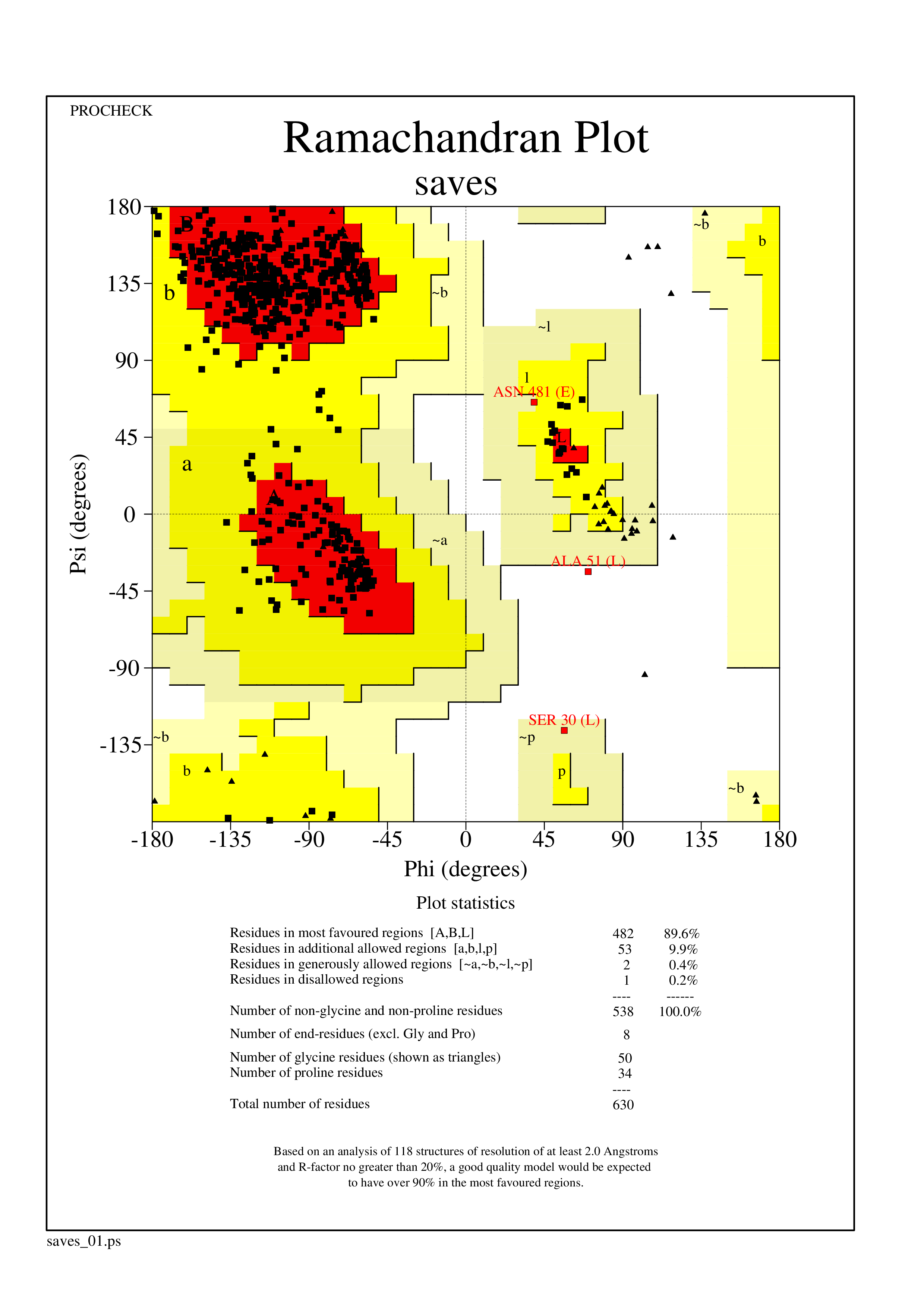


**Figure S1.** Ramachandran Plot for SARS-CoV-2 spike glycoprotein (PDB 7NEH, resolution 1.77 Å)


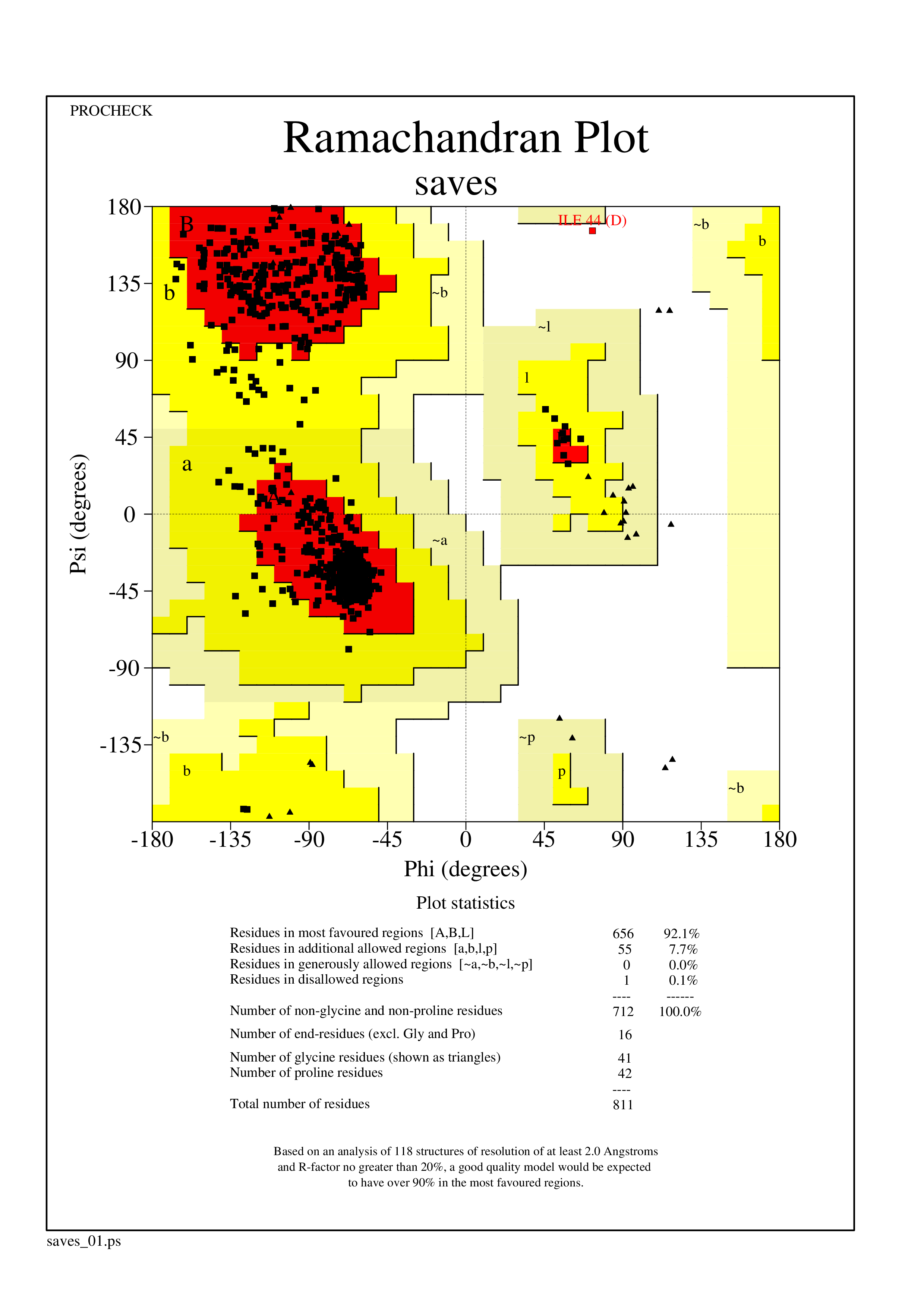


**Figure S2.** Ramachandran Plot for proteins related to the autophagy (PDB 5YEC, resolution 2.15 Å)


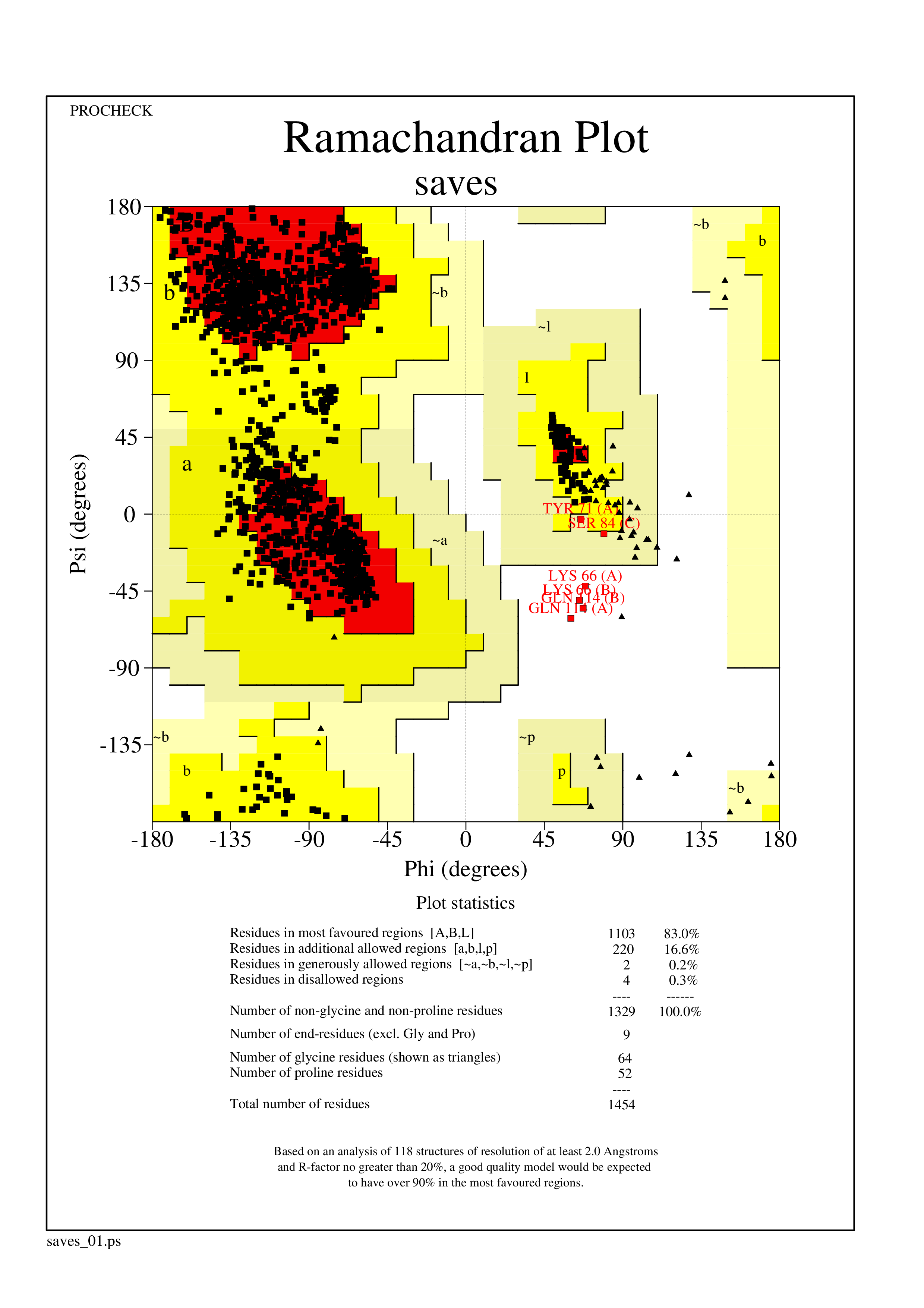


**Figure S3.** Ramachandran Plot for TLR4 (PDB 5IJC, resolution 2.57 Å)


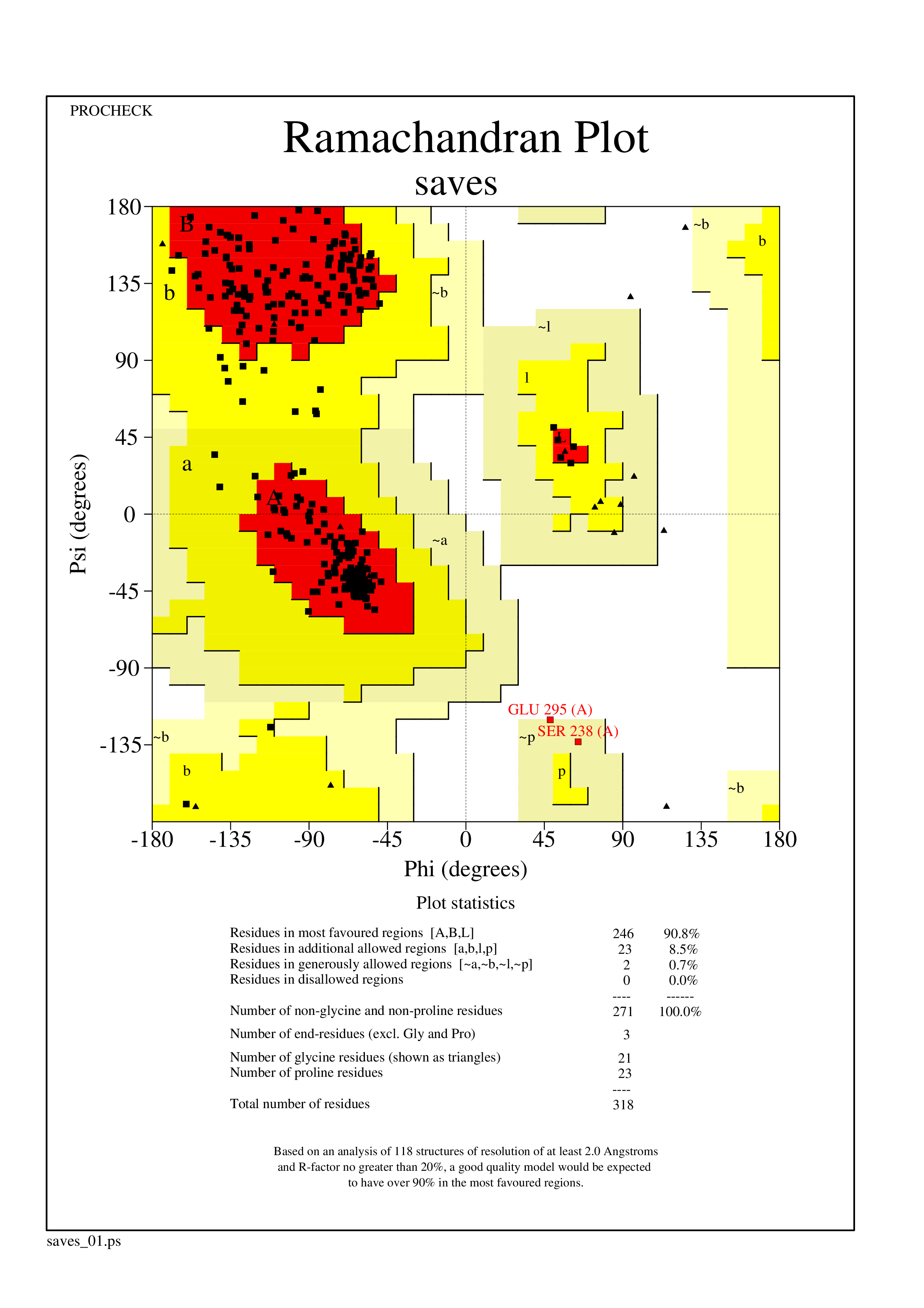


**Figure S4.** Ramachandran Plot for fungal protein (PDB 6QPR, resolution 1.45 Å)

**Table S3.** Molecular docking binding energies of antiviral drugs with SARS-CoV-2 spike glycoprotein (PDB 7NEH)

| **Sr. No.** | **Approved antiviral drugs** | **Binding energy (kcal/mol)** | **RMSD Value**  **(Å)** | **No. of H**  **bonds** | **H-bond with amino acid** |
| --- | --- | --- | --- | --- | --- |
| 1. | Amprenavir | - 7.2 | 1.110 | 1 | SER 399 |
| 2. | Atazanavir | - 8.4 | 1.115 | 4 | THR 345, ASN 354 |
| 3. | Darunavir | - 7.6 | 2.110 | 4 | ALA 397, SER 399, ARG 346 |
| 4. | Fosemprenavir | - 7.1 | 1.153 | 1 | ARG 346 |
| 5. | Indianvir | - 8.0 | 1.321 | 1 | GLU 340 |
| 6. | Lopinavir | - 6.9 | 1.143 | 3 | ARG 346, THR 345 |
| 7. | Nelfinavir | - 7.5 | 2.220 | 2 | THR 345, ARG 346 |
| 8. | Ritonavir | - 7.6 | 1.196 | 1 | ARG 355 |
| 9. | Saquinavir | - 8.0 | 1.285 | 3 | ARG 355, PHE 464, TYR 396 |
| 10. | Tipranavir | - 7.8 | 1.392 | 4 | ASN 334, SER 359, ARG 357 |
| 11. | Delaviridine | - 6.8 | 2.055 | 1 | GLU 516 |
| 12. | Doravirine | - 7.2 | 1.335 | 3 | ASN 354, ARG 355, ARG 466 |
| 13. | Efavirenz | - 7.6 | 1.309 | 2 | SER 399 |
| 14. | Etavirine | - 6.9 | 1.285 | 1 | SER 399 |
| 15. | Nevirapine | - 5.9 | 1.299 | 2 | ALA 348, SER 399 |
| 16. | Rilpivirine | - 6.8 | 1.160 | 1 | ARG 355 |
| 17. | Abacavir | - 5.5 | 1.182 | 1 | GLU 340 |
| 18. | Didanosine | - 5.9 | 1.134 | 4 | THR 345, ARG 346, ALA 397, SER 399 |
| 19. | Emtricitabine | - 5.2 | 1.822 | 6 | ARG 346, SER 399 |
| 20. | Lamivudine | - 5.4 | 2.043 | 6 | ARG 355, THR 430, SER 514, PHE 515 |
| 21. | Stavudine | - 5.5 | 1.358 | 6 | ARG 346, ALA 348, ASN 354, SER 399 |
| 22. | Tenofovir | - 5.9 | 2.118 | 7 | THR 345, ARG 346, ASN 354, SER 399 |
| 23. | Zidovudine | - 5.8 | 1.161 | 4 | THR 345, ARG 346, SER 399 |
| 24. | Bictegravir | - 7.0 | 2.03 | 2 | GLU 340, ARG 346 |
| 25. | Dolutegravir | - 7.2 | 1.211 | 4 | SER 399, ASN 354, ALA 348, ARG 346 |
| 26. | Elvitegravir | - 7.3 | 1.225 | 6 | ARG 346, THR 345, SER 399, ALA 348 |
| 27. | Raltegravir | - 7.1 | 1.429 | 2 | ARG 355 |
| 28. | Fostemsavir | - 6.5 | 1.263 | 3 | ASN 354, ARG 355. |
| 29. | Danoprevir | - 8.2 | 1.090 | 3 | ASN 354, ARG 357, TYR 396. |
| 30. | Grazoprevir | - 7.0 | 1.142 | 1 | LYS 356 |
| 31. | Paritaprevir | - 8.7 | 1.382 | 4 | ARG 357, TYR 396, ARG 355, ASN 354 |
| 32. | Simeprevir | - 7.9 | 1.213 | 3 | ARG 355 |
| 33. | Daclatasvir | - 7.5 | 1.159 | 1 | ASN 334 |
| 34. | Ledipasvir | - 9.6 | 1.281 | 3 | ASN 354, ARG 355, GLU 516 |
| 35. | Ombitasvir | - 8.3 | 1.159 | 5 | ASN 354, ARG 466 |
| 36. | Elbasvir | - 9.4 | 1.396 | 2 | ASN 354, ARG 355, ARG 466 |
| 37. | Velpatasvir | - 7.3 | 1.182 | 4 | ASN 334, ARG 466, THR 345 |
| 38. | Pibrentasvir | - 9.4 | 1.159 | 3 | GLU 340, ALA 352, ASN 354, ARG 355, ARG 357, ARG 466, |
| 39. | Sofosbuvir | - 6.8 | 1.220 | 3 | ARG 355, GLU 340 |
| 40. | Dasabuvir | - 7.9 | 1.383 | 3 | VAL 341, GLU 340, ALA 397, SER 399. |
| 41. | Baloxavir marboxil | - 8.5 | 1.208 | 3 | TRP 353, ASN 354 |
| 42. | Favipiravir | - 4.4 | 1.118 | 4 | ARG 355, SER 514 |
| 43. | Laninamivir | - 6.7 | 1.129 | 4 | ARG 346, ALA 348, ALA 397, SER 399 |
| 44. | Oseltamivir | - 6.2 | 2.031 | 6 | LYS 356, SER 399, ASN 354, ALA 348 |
| 45. | Peramivir | - 6.4 | 1.119 | 3 | SER 399, ALA 348, ARG 346 |
| 46. | Zanamivir | - 6.6 | 1.181 | 5 | THR 345, ARG 346, SER 399 |
| 47. | Ribavirin | - 5.4 | 1.154 | 5 | ARG 346, ASN 354, ARG 355, SER 399 |
| 48. | Aciclovir | - 5.8 | 2.010 | 4 | THR 345, SER 399 |
| 49. | Brivudine | - 5.9 | 1.146 | 5 | ARG 346, ASN 354, ARG 355 |
| 50. | Famciclovir | - 5.5 | 1.183 | 7 | THR 345, ARG 346, SER 399, ASN 345 |
| 51. | Idoxuridine | - 5.8 | 1.153 | 7 | VAL 341, ARG 355, ASN 354, SER 399, ALA 348 |
| 52. | Penciclovir | - 5.9 | 1.333 | 7 | THR 345, ALA 344, ASN 354, SER 399, ARG 346 |
| 53. | Valaciclovir | - 6.1 | 1.205 | 4 | THR 345, SER 399 |
| 54. | Cidofovir | - 5.1 | 1.892 | 5 | ARG 346, SER 399, THR 345 |
| 55. | Foscarnet | - 3.6 | 1.878 | 4 | SERR 514, ARG 355. |
| 56. | Ganciclovir | - 5.8 | 1.181 | 6 | ARG 346, THR 345, GLU 340, SER 399. |
| 57. | Adefovir | - 5.3 | 1.864 | 5 | THR 345, ARG 346, SER 399 |
| 58. | Besifovir | - 6.1 | 1.342 | 2 | ARG 346, SER 399 |
| 59. | Clevudine | - 6.1 | 1.279 | 6 | VAL 341, ALA 348, ASN 354, SER 399 |
| 60. | Tecovirimat | - 5.8 | 1.172 | 1 | GLU 340 |

**Table S4.** Molecular docking binding energies of antiviral drugs with autophagy receptor (PDB 5YEC)

| **Sr. No.** | **Approved antiviral drugs** | **Binding energy (kcal/mol)** | **RMSD Value**  **(Å)** | **No. of H**  **bonds** | **H-bond with amino acid** |
| --- | --- | --- | --- | --- | --- |
| 1. | Amprenavir | - 6.6 | 2.258 | 2 | GLU 381, ARG 389 |
| 2. | Atazanavir | - 7.5 | 2.358 | 1 | LYS 388 |
| 3. | Darunavir | - 7.5 | 2.374 | 2 | ASN 371,  ARG 389 |
| 4. | Fosemprenavir | - 6.4 | 2.316 | 3 | LYS 377, PRO 378, ARG 389 |
| 5. | Indianvir | - 7.3 | 2.141 | 1 | ARG 389 |
| 6. | Lopinavir | - 8.1 | 2.366 | 1 | ARG 389 |
| 7. | Nelfinavir | - 7.0 | 2.258 | 2 | GLU 381, ALA  385 |
| 8. | Ritonavir | - 6.7 | 2.243 | 1 | GLU 381 |
| 9. | Saquinavir | - 7.9 | 2.214 | 1 | LYS 388 |
| 10. | Tipranavir | - 7.3 | 2.263 | 1 | GLU 381 |
| 11. | Delaviridine | - 6.9 | 2.387 | 1 | GLU 381 |
| 12. | Doravirine | - 6.3 | 2.227 | 1 | LYS 377 |
| 13. | Efavirenz | - 5.9 | 2.258 | 3 | LYS 388, PRO 392 |
| 14. | Etavirine | - 6.0 | 2.267 | 1 | GLY 398 |
| 15. | Nevirapine | - 5.4 | 2.251 | 1 | PRO 392 |
| 16. | Rilpivirine | - 6.6 | 2.277 | 1 | LYS 377 |
| 17. | Abacavir | - 4.9 | 2.325 | 1 | GLY 398 |
| 18. | Didanosine | - 5.3 | 2.449 | 3 | THR 358 |
| 19. | Emtricitabine | - 4.5 | 2.212 | 6 | ASN 371, ASP 374, LYS 377, ARG 389, |
| 20. | Lamivudine | - 4.3 | 2.149 | 5 | LYS 418, ASP 419, ARG 422 |
| 21. | Stavudine | - 4.7 | 2.265 | 2 | ASN 371, LYS 377 |
| 22. | Tenofovir | - 4.8 | 2.249 | 1 | GLU 373 |
| 23. | Zidovudine | - 4.8 | 2.229 | 2 | LYS 377, ARG 389 |
| 24. | Bictegravir | - 6.9 | 2.262 | 1 | LYS 377 |
| 25. | Dolutegravir | - 6.4 | 2.289 | 1 | LYS 377 |
| 26. | Elvitegravir | - 6.2 | 2.228 | 1 | GLU 381 |
| 27. | Raltegravir | - 6.6 | 2.357 | 1 | LYS 388 |
| 28. | Fostemsavir | - 6.1 | 2.433 | 3 | LYS 377, LYS 388 |
| 29. | Danoprevir | - 7.9 | 2.265 | 2 | LYS 377, ARG 389 |
| 30. | Grazoprevir | - 7.3 | 2.359 | 1 | GLU 381 |
| 31. | Paritaprevir | - 8.7 | 1.558 | 4 | GLU 381, ALA 384, ALA 385, LYS 388, ARG 389 |
| 32. | Simeprevir | - 7.8 | 1.898 | 1 | LYS 377 |
| 33. | Daclatasvir | - 8.2 | 2.340 | 1 | ARG 422 |
| 34. | Ledipasvir | - 9.7 | 2.213 | 2 | GLU 381, ALA 385, LYS 388, LYS 400, LYS 418, ARG 422 |
| 35. | Ombitasvir | - 7.4 | 2.294 | 3 | GLY 398, LYS 377, ARG 422 |
| 36. | Elbasvir | - 9.0 | 2.231 | 3 | GLY 376, LYS 377, ALA 384, ALA 385, LYS 388, ALA 396, GLY 398 |
| 37. | Velpatasvir | - 8.2 | 2.259 | 1 | LYS 377 |
| 38. | Pibrentasvir | - 7.5 | 1.979 | 2 | PRO 378, LYS 400 |
| 39. | Sofosbuvir | - 6.4 | 2.246 | 1 | LYS 377 |
| 40. | Dasabuvir | - 6.9 | 2.145 | 3 | ALA 385, ARG 389 |
| 41. | Baloxavir marboxil | - 6.6 | 2.324 | 3 | LYS 388, PRO 392 |
| 42. | Favipiravir | - 3.7 | 2.312 | 4 | GLN 367, ASN 363, LYS 379 |
| 43. | Laninamivir | - 5.1 | 2.220 | 4 | ARG 422, LYS 418 |
| 44. | Oseltamivir | - 4.9 | 2.363 | 5 | LYS 418, ASP 419, ARG 422 |
| 45. | Peramivir | - 4.8 | 2.012 | 2 | GLU 381, GLY 398 |
| 46. | Zanamivir | - 5.1 | 2.339 | 3 | LYS 377, ARG 389 |
| 47. | Ribavirin | - 4.9 | 2.312 | 6 | ARG 389, ASN 371, GLU 373, LYS 377 |
| 48. | Aciclovir | - 4.4 | 2.284 | 5 | SER 402, LYS 418, ASP 419, ARG 422 |
| 49. | Brivudine | - 4.8 | 2.275 | 1 | GLY 398 |
| 50. | Famciclovir | - 4.6 | 2.285 | 2 | PRO 378, GLY 398 |
| 51. | Idoxuridine | - 4.9 | 2.417 | 4 | LYS 418, ASP 419, SER 402 |
| 52. | Penciclovir | - 4.5 | 2.292 | 3 | LYS 377, PRO 378, GLU 381 |
| 53. | Valaciclovir | - 5.1 | 2.316 | 3 | GLY 398, THR 397, ALA 396 |
| 54. | Cidofovir | - 4.7 | 2.342 | 4 | LYS 400, LYS 418, ARG 422 |
| 55. | Foscarnet | - 5.4 | 2.458 | 9 | ARG 341, ASN 371, ARG 389 |
| 56. | Ganciclovir | - 4.4 | 2.348 | 6 | SER 402, LYS 418, ASP 419 |
| 57. | Adefovir | - 4.7 | 2.190 | 4 | SER 402, LYS 418, ARG 422 |
| 58. | Besifovir | - 4.7 | 2.189 | 4 | LYS 418, ARG 422 |
| 59. | Clevudine | - 4.7 | 2.313 | 2 | LYS 377, ARG 389 |
| 60. | Tecovirimat | - 6.2 | 2.314 | 3 | GLY 398, LYS 400, ARG 422 |

**Table S5.** Molecular docking binding energies of antiviral drugs with TLR4 (PDB 5IJC)

| **Sr. No.** | **Approved antiviral drugs** | **Binding energy (kcal/mol)** | **RMSD Value**  **(Å)** | **No. of H**  **bonds** | **H-bond with amino acid** |
| --- | --- | --- | --- | --- | --- |
| 1. | Amprenavir | - 9.5 | 1.523 | 2 | ILE 80, VAL 82, PHE 121 |
| 2. | Atazanavir | - 11.0 | 1.582 | 3 | VAL 61, VAL 82, PHE 121 |
| 3. | Darunavir | - 10.4 | 1.591 | 1 | ARG 90 |
| 4. | Fosemprenavir | - 9.8 | 1.536 | 1 | ARG 90 |
| 5. | Indianvir | - 11.3 | 1.621 | 0 | VAL 82, PHE 121, PRO 127, ILE 153, PHE 438, SER 439 |
| 6. | Lopinavir | -10.2 | 1.635 | 2 | PHE 126, PHE 438 |
| 7. | Nelfinavir | - 11.0 | 1.601 | 1 | SER 439 |
| 8. | Ritonavir | - 9.1 | 1.611 | 2 | ARG 90 |
| 9. | Saquinavir | - 11.5 | 1.592 | 0 | ILE 52, LEU 54, ILE 80, VAL 82, PHE 121, ILE 124, PHE 126,PRO 127,PHE 438, |
| 10. | Tipranavir | - 10.6 | 1.703 | 1 | PHE 126 |
| 11. | Delaviridine | - 9.2 | 1.621 | 2 | ARG 90, GLU 92 |
| 12. | Doravirine | - 9.1 | 1.587 | 3 | MET 412, ARG 434 |
| 13. | Efavirenz | - 8.9 | 1.642 | 2 | SER 439 |
| 14. | Etavirine | - 8.9 | 1.642 | 1 | MET 412 |
| 15. | Nevirapine | - 7.8 | 1.638 | 2 | ILE 124, PHE 438 |
| 16. | Rilpivirine | - 8.6 | 1.561 | 3 | GLY 387, SER 390, SER 392 |
| 17. | Abacavir | - 7.2 | 1.637 | 2 | VAL 82, ILE 124 |
| 18. | Didanosine | - 7.0 | 1.573 | 1 | ARG 90 |
| 19. | Emtricitabine | - 5.9 | 1.567 | 2 | LEU 125, SER 439 |
| 20. | Lamivudine | - 6.0 | 1.595 | 4 | ARG 90, SER 413, SER 439, |
| 21. | Stavudine | - 6.5 | 1.616 | 3 | ARG 90, SER 439 |
| 22. | Tenofovir | - 6.9 | 1.611 | 1 | SER 439 |
| 23. | Zidovudine | - 7.2 | 1.643 | 2 | SER 439 |
| 24. | Bictegravir | - 10.0 | 1.515 | 2 | PHE 126, CYS 133 |
| 25. | Dolutegravir | - 9.5 | 1.632 | 2 | ARG 90, LEU 87 |
| 26. | Elvitegravir | - 10.2 | 1.617 | 3 | SER 413, ARG 434 |
| 27. | Raltegravir | - 8.9 | 1.580 | 1 | SER 413 |
| 28. | Fostemsavir | - 9.1 | 1.531 | 2 | PHE 126, GLU 92 |
| 29. | Danoprevir | - 9.8 | 1.601 | 2 | PHE 76, SER 413 |
| 30. | Grazoprevir | - 10.4 | 1.771 | 1 | TYR 102 |
| 31. | Paritaprevir | - 10.8 | 1.595 | 2 | GLU 92, ARG 434 |
| 32. | Simeprevir | - 10.4 | 1.459 | 2 | PHE 121, GLU 122 |
| 33. | Daclatasvir | - 8.2 | 1.626 | 2 | HIS 92, ASP 101, LYS 433 |
| 34. | Ledipasvir | - 11.6 | 1.584 | 3 | LYS 89, ARG 90, PHE 121, ILE 124, PHE 126, ARG 434, GLU 437 |
| 35. | Ombitasvir | - 10.4 | 1.589 | 1 | ARG 434 |
| 36. | Elbasvir | - 8.9 | 1.651 | 1 | LYS 263 |
| 37. | Velpatasvir | - 7.0 | 1.576 | 2 | ILE 52, ARG 434 |
| 38. | Pibrentasvir | - 7.9 | 1.662 | 2 | ARG 90, GLU 92 |
| 39. | Sofosbuvir | - 9.3 | 1.587 | 1 | SER 439 |
| 40. | Dasabuvir | - 10.3 | 1.592 | 1 | PHE 438 |
| 41. | Baloxavir marboxil | - 10.9 | 1.632 | 1 | SER 413 |
| 42. | Favipiravir | - 5.1 | 1.529 | 4 | SER 439, LEU 125, ASN 415 |
| 43. | Laninamivir | - 7.8 | 1.549 | 1 | PHE 126 |
| 44. | Oseltamivir | - 7.8 | 1.627 | 2 | LEU 125, ASN 415 |
| 45. | Peramivir | - 8.0 | 1.739 | 1 | SER 439 |
| 46. | Zanamivir | - 7.7 | 1.648 | 5 | LEU 125, ASN 415, SER 439 |
| 47. | Ribavirin | - 6.3 | 1.569 | 6 | LEU 125, ASN 415, SER 439 |
| 48. | Aciclovir | - 6.5 | 1.660 | 4 | LEU 125, ASN 415, SER 439 |
| 49. | Brivudine | - 7.5 | 1.576 | 4 | ARG 90, SER 413 |
| 50. | Famciclovir | - 6.7 | 1.636 | 2 | ARG 90, SER 439 |
| 51. | Idoxuridine | - 6.8 | 1.505 | 5 | ASN 415, SER 439 |
| 52. | Penciclovir | - 6.8 | 1.599 | 5 | LEU 125, ASN 415, SER 439 |
| 53. | Valaciclovir | - 7.6 | 1.636 | 4 | LEU 125, ASN 415, SER 439 |
| 54. | Cidofovir | - 6.4 | 1.595 | 2 | LEU 87, VAL 82 |
| 55. | Foscarnet | - 3.9 | 1.603 | 8 | VAL 435, ARG 434, LEU 432, LYS 458, ASN 456 |
| 56. | Ganciclovir | - 6.5 | 1.659 | 5 | LEU 125, ASN 415, SER 439 |
| 57. | Adefovir | - 6.0 | 1.613 | 1 | ARG 90 |
| 58. | Besifovir | - 7.4 | 1.616 | 1 | SER 439 |
| 59. | Clevudine | - 6.9 | 1.597 | 4 | LEU 125, ASN 415, SER 439 |
| 60. | Tecovirimat | - 9.0 | 1.590 | 1 | ILE 124 |

**Table S6.** Molecular docking binding energies of antifungal drugs with target protein of Rhizomucor miehei (PDB 6QPR)

| **Sr. No.** | **Approved antiviral drugs** | **Binding energy (kcal/mol)** | **RMSD Value**  **(Å)** | **No. of H**  **bonds** | **H-bond with amino acid** |
| --- | --- | --- | --- | --- | --- |
| 1. | Amphotericin | - 5.5 | 2.012 | 1 | PRO 70 |
| 2. | Anidulagin | - 4.4 | 1.983 | 1 | ASP 88 |
| 3. | Caspofungin | - 6.5 | 1.932 | 3 | GLU 295, ASN 321, SER 353 |
| 4. | Econazole | - 7.2 | 2.102 | 1 | PRO 68, LEU 69, GLY 78 |
| 5. | Fluconazole | - 5.4 | 1.832 | 2 | ASN 294, GLY 356 |
| 6. | Isavuconazole | - 6.7 | 1.891 | 1 | ARG 65 |
| 7. | Itraconazole | - 8.0 | 1.923 | 1 | SER 85, GLU 295, LEU 349 |
| 8. | Luliconazole | - 5.7 | 1.813 | 1 | SER 341 |
| 9. | Micafungin | - 4.8 | 1.804 | 1 | ASP 88 |
| 10. | Miconazole | - 6.8 | 1.793 | 1 | ASP 88 |
| 11. | Nystatin | - 2.9 | 0.876 | 2 | ASP 72, SER 71, |
| 12. | Posaconazole | - 5.6 | 2.032 | 1 | ASN 82 |
| 13. | Voriconazole | - 5.4 | 1.617 | 1 | ASN 82 |
| 14. | Ipconazole | - 6.3 | 1.793 | 1 | THR 75 |
| 15. | Isoconazole | - 6.7 | 1.472 | 1 | TRP 317 |
| 16. | Sulconazole | - 6.8 | 0.807 | 1 | ILE 342 |
| 17. | Terconazole | - 6.9 | 0.753 | 1 | ASP 350 |
| 18. | Tioconazole | - 6.2 | 0.678 | 1 | ASP 337 |
| 19. | Clotrimazole | - 4.8 | 1.322 | 1 | ASP 88 |
| 20. | Terbinafine | - 5.1 | 1.432 | 1 | TYR 86 |
